# Supplementary material for: Biomarkers of Frailty in Patients with Advanced Chronic Liver Disease Undergoing a Multifactorial Intervention Consisting of Home Exercise, Branched-Chain Amino Acids, and Probiotics
Source: Biomolecules. 2024 Nov 6;14(11):1410. doi: 10.3390/biom14111410 (PMC11592179; doi:10.3390/biom14111410)
Supplement: Supplementary file 1 [file biomolecules-14-01410-s001.zip › biomolecules-3240750-supplementary.pdf]

SUPPLEMENTARY INFORMATION

SUPPLEMENTARY PATIENTS AND METHODS

Evaluation of frailty: Liver Frailty Index (LFI)

The LFI includes handgrip strength, the ability to get up from and sit on a chair (timed chair stands), and balance. Detailed instructions regarding performance of the LFI can be found at https://liverfrailtyindex.ucsf.edu/. The LFI has been validated in patients with cirrhosis and is currently the most widely accepted tool to evaluate frailty in these patients. The LFI classifies patients with a score of >4.4 as frail, those with score of 4.4-3.2 as pre-frail, and those with a score of <3.2 as robust. A clinically significant improvement in LFI is considered moderate if ≥0.2 and substantial if ≥0.5 [5,7,24].

Muscle function and body composition assessed by handgrip strength, electrical bioimpedance and ultrasound

Muscle function was evaluated by handgrip strength assessed by a dynamometer (KERN MAP 80K1, Akern) following the manufacturer's instructions. We used the total body impedance analyzer BIA 101 at a signal frequency of 50 kHz and the software BodyGram PRO V.3.0 (Akern, Florence, Italy) to calculate the phase angle and the estimated body compartments adjusting for age, sex, weight and height. Measurements were made in supine position with 4 conventional electrodes: 2 on the wrist and 2 on the ipsilateral foot. Indications before the test were no food or drink in the previous 4 hours, no exercise in the previous 12 hours, an empty bladder, and removal of any jewellery and clothing with metallic elements [22].

Moreover, in patients in the intervention group, with the patient in supine position and hip and knee extended, medium thigh circumference was determined using a measuring tape and thickness of the quadriceps was evaluated by ultrasound (Philips EPIQ Elite: mode B, 8MHz, linear transductor) at the medium thigh and at the limit between the upper third and the medium third of the thigh.

Multifactorial intervention

Exercise at home: The exercise programme at home was based on previous reports in patients with cirrhosis [56] and our experience with in-hospital exercise programmes in these patients [10,57]. The programme was especially aimed at rehabilitation of the frail patient and was multicomponent. It consisted of aerobic and anaerobic exercise by pedalling (cardiovascular resistance), resistance exercises with dumbbells and elastic bands (muscular strength), flexibility training by stretching, and coordination and balance exercises. Patients performed 3 sessions of 20-30 minutes per week, with progressive increases for up to 45-60 minutes according to tolerance. Each patient in the intervention group was provided with a pedalling or mini-static bicycle (mini-bike DOMYOS 100, Decathlon), weights (dumbbells) and elastic bands (Decathlon), and a watch heart rate monitor (ONRHYTHM 110-Kalenji).

At the beginning of the study patients were visited by a physician specialized in physical medicine and a physiotherapist. At this visit, they received instruction in the exercise programme. This was individualized according to their physical condition and they were given the necessary material and written information

and instructions regarding the programme. To prevent variceal bleeding, exercises that could significantly increase intraabdominal pressure were avoided. The visit was repeated at 6 months and at the end of the study to check compliance, evaluate possible problems with the programme and review the exercises.

Multistrain probiotic: Marketed under the brand Vivomixx® (De Simone Formulation) (Mendes SA, Lugano, Switzerland) in Europe and Visbiome® (ExeGi Pharmaceuticals, Rockville, MD) in the USA, the multistrain probiotic was a mixture containing the 8 following bacterial strains*: Streptococcus thermophilus* DSM 24731®/NCIMB 30438, *Bifidobacterium breve* DSM 24732®/NCIMB 30441*, Bifidobacterium longum* DSM 24736®/NCIMB 30435 (reclassified *B. lactis*), *Bifidobacterium infantis* DSM 24737®/NCIMB 30436 (reclassified *B. lactis*), *Lactobacillus acidophilus* DSM 24735®/NCIMB 30442*, Lactobacillus plantarum* DSM 24730®/NCIMB 30437*, Lactobacillus paracasei* DSM 24733®/NCIMB 30439*, Lactobacillus delbrueckii* subsp. *bulgaricus* DSM 24734®/NCIMB 30440 (reclassified *L. helveticus*). Patients from the intervention group took one sachet of 4.4 g every 12 hours ($450x10^9$ live bacteria per sachet) throughout the study. The excipient was maltose. The probiotic mixture was manufactured by Danisco-DuPont (Madison, WI, USA). Patients were instructed to keep the study product at 4ºC in the refrigerator at home and to take the sachets diluted in one glass of water, milk, or juice at room temperature. We chose this multistrain probiotic because previous studies in patients with cirrhosis have shown its efficacy in preventing hepatic encephalopathy and improving cognitive function and risk of falls [17,58].

Branched-chain amino acids (BCAA): In addition to the exercise programme and the probiotic, patients in the intervention group received BCAA supplements (L-leucine, isoleucine and valine in powder with a 8:1:1 ratio in favor of L-leucine) 10 g 30 minutes before each exercise session throughout the study to enhance the effect of exercise on muscle function and mass. We chose this dose based on previous data in the literature [10,59].

Weekly phone calls: Each week, a nurse from the research team phoned participants in the intervention group to ensure adherence to the multifactorial intervention and to detect possible complications.

Malondialdehyde (MDA) determination

MDA in plasma was determined using a spectrophotometric method. First, 40% TCA was added to the plasma for MDA precipitation. Subsequently, a thiobarbituric acid (TBA) solution was added and the mixture was heated to 100°C for 45 minutes to allow the reaction between MDA and TBA, forming a pink complex. The samples were then cooled to room temperature, and the absorbance of the MDA-TBA complex was measured at 532 nm. A TPE solution was used to prepare the standard curve [60].

SUPPLEMENTARY REFERENCES

56. Kruger, C.; McNeely, M.L.; Bailey, R.J.; Yavari, M.; Abraldes, J.G.; Carbonneau, M.; Newnham, K.; DenHeyer, V.; Ma, M.; Thompson, R.; et al. Home exercise training improves exercise capacity in cirrhosis patients: role of exercise adherence. *Sci. Rep.* **2018**, *8*, 99.
57. Román, E.; García-Galcerán, C.; Torrades, T.; Herrera, S.; Marín, A.; Doñate, M.; Alvarado-Tapias, E.; Malouf, J.; Nácher, L.; Serra-Grima, R.; et al. Effects of an exercise programme on functional capacity, body composition and risk of falls in patients with cirrhosis: a randomized clinical trial. *PLoS. One.* **2016**, *24*, e0151652.
58. Dhiman, R.K.; Rana, B.; Agrawal, S.; Garg, A.; Chopra, M.; Thumburu, K.K.; Khattri, A.; Malhotra, S.; Duseja, A.; Chawla, Y.K. Probiotic VSL#3 reduces liver disease severity and hospitalization in patients with cirrhosis: a randomized, controlled trial. *Gastroenterology.* **2014**, *147*, 1327-1337.
59. Gluud, L.L.; Dam, G.; Les, I.; Marchesini, G.; Borre, M.; Aagaard, N.K.; Vilstrup, H. Branched-chain amino acids for people with hepatic encephalopathy. *Cochrane Database Syst. Rev.* 2017, *18*, 5:CD001939.
60. Sánchez, E.; Nieto, J.C.; Vidal, S.; Santiago, A.; Martinez, X.; Sancho, F.J.; Sancho-Bru, P.; Mirelis, B.; Corominola, H.; Juárez, C.; et al. Fermented milk containing *Lactobacillus paracasei* subsp. *paracasei* CNCM I-1518 reduces bacterial translocation in rats treated with carbon tetrachloride. Sci. Rep. **2017**, *7*, 45712.

SUPPLEMENTARY TABLES

Supplementary Table S1. Clinical meaning in the context of advanced chronic liver disease of the biomarkers included in the study.

Biomarker	Clinical meaning
LFI	Increase with frailty, worse prognosis
Phase angle	Decrease with impairment in body composition, worse prognosis
CRP	Increase in inflammation, worse prognosis
LBP	Increase in bacterial translocation
IL-6	Increase in inflammation, frailty
TNF-α	Increase in inflammation
TNFsR1	Increase in inflammation, frailty
sCD63	Increase in macrophage activation, inflammation, frailty
sMR	Increase in macrophage activation, inflammation
IgA	Decrease in impaired gastrointestinal mucosa defense
GDF-15	Increase in frailty
AHSG	Decrease in frailty
FGF-21	Increased in sarcopenia in cirrhosis, metabolic syndrome
ccK18	Increase in hepatocyte damage
mtDNA	Increase with mitochondrial health and decrease in frailty
Copeptin	Increase in hemodynamic impairment, worse prognosis
LRG1	Increase in frailty
Myostatin	Increase in sarcopenia
Cystatin-C	Increase in renal failure
Vitamin D	Decrease in frailty and sarcopenia
Resistin	Increase in metabolic syndrome
MDA	Increase in oxidative stress
Claudin-3	Increase in intestinal barrier impairment
uNGAL	Increase in worse prognosis and acute tubular necrosis

LFI: Liver Frailty Index; CRP: c-reactive protein; LBP: lipopolysaccharide binding protein; TNFsR1: tumor necrosis factor soluble receptor 1; sCD163: soluble CD163; sMR: soluble mannose receptor; GDF-15: growth differentiation factor 15; AHSG: alpha-2-Heremans-Schmid glycoprotein; FGF-21: fibroblast growth factor 21; ccK18: caspase-cleaved keratin 18; mtDNA: mitochondrial DNA; LRG1: leucine rich alpha-2-glycoprotein 1; MDA: malondialdehyde; uNGAL: urinary neutrophil gelatinase-associated lipocalin.

Supplementary Table S2. Results of LFI and biomarkers at baseline and at 3, 6 ,9 and 12 months in the control group and the intervention group. Results expressed as mean±SEM.

	Control group					Intervention group				
	Baseline	3 months	6 months	9 months	12 months	Baseline	3 months	6 months	9 months	12 months
LFI[1]	4.07±0.12	4.01±0.19	3.96±0.11	3.96±0.14	3.91±0.14	4.00±0.08	3.62±0.10	3.66±0.10	3.47±0.13	3.31±0.12
CRP[2] (mg/L)	4.19±0.87	3.09±0.44	8.74±3.4	6.19±2.32	5.24±1.29	3.66±0.84	2.53±0.45	2.44±0.35	3.82±1.80	2.05±0.42
LBP[3] (µg/mL)	7.39±0.18	7.14±0.18	6.75±0.15	6.77±0.22	6.58±0.21	7.25±0.21	6.46±0.22	6.16±0.26	5.75±0.23	5.32±0.23
IL-6 (pg/mL)	24.7±9.7	14.4±6.5	30.5±11.6	26.8±13.1	13.2±5.1	24.3±14.0	32.2±24.6	36.8±19.2	13.8±12.3	5.7±4.2
TNF-α (pg/mL)	5.3±2.3	5.3±1.8	14.7±7.1	4.1±2.4	6.3±2.7	5.5±3.9	7.9±4.8	9.6±5.1	1.3±0.9	11.8±6.8
TNFsR1 (ng/mL)	1.17±0.09	1.12±0.08	1.27±0.11	1.42±0.10	1.18±0.11	1.18±0.11	1.28±0.12	1.50±0.20	1.14±0.18	1.11±0.14
sCD63[4] (mg/L)	6.93±0.30	6.76±0.26	6.16±0.20	5.67±0.18	4.4±0.18	6.24±0.23	5.82±0.24	5.67±0.26	5.05±0.26	2.99±0.08
sMR[5] (mg/L)	0.58±0.03	0.54±0.03	0.47±0.02	0.41±0.02	0.36±0.02	0.51±0.03	0.47±0.03	0.41±0.02	0.33±0.02	0.22±0.01
IgA (mg/mL)	12.0±1.8	13.7±1.8	11.8±1.5	9.8±1.7	10.3±1.6	12.5±1.8	11.4±1.5	12.4±1.9	11.4±2.5	8.9±1.4
GDF-15 (ng/mL)	3.45±0.43	2.77±0.42	4.12±0.10	3.03±0.31	2.78±0.26	3.07±0.69	2.47±0.48	2.71±0.54	2.48±0.83	1.97±0.58
AHSG (µg/mL)	343±37	296±36	271±30	307±56	337±27	485±92	406±56	387±53	426±67	404±56
FGF-21 (pg/mL)	334±83	207±41	251±59	197±48	249±65	206±51	225±65	176±42	101±17	123±18
ccK18[6] (U/L)	721±30	678±27	632±26	564±31	478±37	679±33	583±24	491±25	360±44	272±22
mtDNA (copies/µL)	39.7±3.3	50.8±3.6	65.1±4.0	79.7±4.2	99.5±2.2	43.8±3.4	54.8±2.7	64.0±3.1	82.4±3.2	130.0±5.5
Copeptin (pg/mL)	422±113	332±106	363±101	350±123	296±69	265±75	306±101	284±64	289±50	275±73
LRG1 (µg/mL)	277±81	211±50	260±71	191±80	178±47	269±60	179±47	170±45	222±80	219±66
Myostatin (ng/mL)	1.11±0.18	1.10±0.16	0.98±0.17	0.87±0.15	1.16±0.19	0.79±0.09	0.85±0.07	0.88±0.09	0.78±0.11	0.68±0.06
Cystatin C (mg/L)	1.10±0.06	1.09±0.05	1.05±0.06	1.25±0.07	1.11±0.07	1.16±0.06	1.15±0.05	1.19±0.06	1.05±0.07	1.06±0.06
Vitamin D (nmol/L)	55.3±13.9	64.1±12.4	63.1±13.8	52.0±13.8	54.0±11.2	42.9±9.0	47.3±9.2	35.7±9.4	47.5±16.7	50.4±12.7
Resistin (ng/mL)	40.7±4.1	40.5±5.6	42.7±5.9	44.9±1.3	40.7±4.5	42.7±3.7	47.8±4.7	48.6±4.8	41.2±3.9	38.2±3.8
MDA (nmol/L)	4.0±0.9	2.5±0.2	3.4±0.4	3.2±0.5	3.8±0.6	3.3±0.6	3.4±0.7	3.1±0.4	2.8±0.2	3.6±0.8

[1] p=0.019, [2] p=0.029, [3] p= 0.018, [4] p=0.01, [5] p=0.058, [6] p=0.005 between intervention group and control group using lineal mixed models.
P NS between the two groups in the remaining biomarkers.

LFI: Liver Frailty Index; CRP: c-reactive protein; LBP: lipopolysaccharide binding protein; TNFsR1: tumor necrosis factor soluble receptor 1; sCD163: soluble CD163; sMR: soluble mannose receptor; GDF-15: growth differentiation factor 15; AHSG: alpha-2-Heremans-Schmid glycoprotein; FGF-21: fibroblast growth factor 21; ccK18: caspase-cleaved keratin 18; mtDNA: mitochondrial DNA; LRG1: leucine rich alpha-2-glycoprotein 1; MDA: malondialdehyde.

Supplementary Table S3. Delta change between baseline and 3, 6, 9 and 12 months in all the metabolites identified using ^{1}H-NMR in the control group (upper row) and the intervention group (lower row). Results are expressed in mmol/L as mean ± SEM. P values between the control group and the intervention group. P values in bold indicate statistical significance.

Molecule	Δ 3 mo. - baseline	Δ 6 mo. - baseline	Δ 9 mo. - baseline	Δ 12 mo. - baseline	p
Formate	-2.81E-03 ± 9.16E-04<br>-7.26E-04 ± 5.46E-04	-4.59E-03 ± 4.13E-03<br>-1.27E-03 ± 9.93E-04	-4.34E-03 ± 2.51E-03<br>-2.49E-03 ± 1.25E-03	-2.56E-03 ± 2.14E-03<br>-2.02E-03 ± 1.23E-03	0.183
Phenylalanine	1.28E-03 ± 2.52E-03<br>2.82E-03 ± 2.31E-03	3.45E-03 ± 7.65E-03<br>2.60E-03 ± 2.39E-03	4.65E-04 ± 4.01E-03<br>-2.26E-03 ± 4.76E-03	3.80E-03 ± 2.42E-03<br>3.73E-04 ± 2.61E-03	0.834
Histidine	-8.49E-04 ± 1.28E-03<br>1.22E-03 ± 9.56E-04	-7.68E-04 ± 3.66E-03<br>3.82E-04 ± 9.26E-04	-6.30E-04 ± 1.69E-03<br>-1.77E-04 ± 2.39E-03	2.04E-04 ± 1.13E-03<br>-5.39E-04 ± 8.27E-04	0.248
Tyrosine	-6.21E-04 ± 1.39E-03<br>-8.97E-05 ± 8.91E-04	-2.82E-03 ± 3.33E-03<br>4.94E-05 ± 9.86E-04	-1.75E-03 ± 1.21E-03<br>-1.06E-03 ± 1.83E-03	-2.13E-03 ± 8.45E-04<br>-1.40E-03 ± 7.94E-04	0.102
Mannose	8.66E-04 ± 9.88E-04<br>-1.01E-03 ± 1.37E-03	5.89E-04 ± 2.24E-03<br>-7.95E-04 ± 8.92E-04	1.52E-03 ± 1.41E-03<br>-4.46E-04 ± 1.78E-03	2.48E-04 ± 8.08E-04<br>-3.45E-06 ± 8.03E-04	0.095
Lactate	4.54E-03 ± 2.19E-02<br>9.29E-04 ± 2.36E-02	1.38E-02 ± 7.43E-02<br>-2.92E-02 ± 2.57E-02	-6.03E-03 ± 2.91E-02<br>7.77E-03 ± 4.90E-02	3.45E-02 ± 4.02E-02<br>2.55E-02 ± 3.36E-02	0.694
Creatinine	1.46E-03 ± 5.22E-04<br>-7.79E-04 ± 5.01E-04	7.00E-04 ± 9.84E-04<br>-1.08E-03 ± 5.15E-04	-7.43E-04 ± 7.83E-04<br>-5.42E-04 ± 6.26E-04	1.19E-03 ± 3.77E-04<br>-7.87E-05 ± 7.10E-04	**0.001**
Serine	3.57E-04 ± 1.93E-03<br>5.02E-04 ± 3.54E-03	-2.85E-03 ± 1.06E-02<br>-2.66E-04 ± 3.63E-03	-9.91E-04 ± 3.13E-03<br>-8.59E-04 ± 2.78E-03	-7.31E-04 ± 2.58E-03<br>2.52E-03 ± 2.87E-03	0.561
Betaine	4.27E-03 ± 2.66E-03<br>6.01E-04 ± 3.64E-03	8.15E-05 ± 2.87E-03<br>1.23E-03 ± 3.29E-03	5.70E-03 ± 2.94E-03<br>9.09E-04 ± 4.00E-03	3.34E-03 ± 2.01E-03<br>-1.74E-03 ± 2.39E-03	0.141
Glycerol	-6.39E-03 ± 6.09E-03<br>2.64E-01 ± 2.97E-01	1.27E+00 ± 2.21E+00<br>2.08E-03 ± 9.94E-03	1.86E-02 ± 2.49E-02<br>3.92E-02 ± 5.05E-02	5.34E-03 ± 6.26E-03<br>1.82E-02 ± 1.56E-02	0.559
myo-Inositol	-5.75E-04 ± 1.24E-03<br>2.28E-04 ± 1.05E-03	-1.23E-04 ± 2.47E-03<br>-2.79E-04 ± 4.99E-04	-5.10E-04 ± 1.23E-03<br>4.72E-04 ± 1.22E-03	9.30E-04 ± 1.28E-03<br>7.81E-05 ± 9.50E-04	0.459
Threonine	-1.97E-03 ± 3.17E-03<br>5.14E-03 ± 4.86E-03	3.22E-02 ± 5.56E-02<br>1.51E-03 ± 3.84E-03	1.84E-04 ± 6.15E-03<br>1.68E-03 ± 5.92E-03	-1.89E-03 ± 3.24E-03<br>5.38E-03 ± 2.77E-03	0.654
Glycine	8.13E-03 ± 5.20E-03<br>1.06E-02 ± 5.16E-03	6.55E-03 ± 1.09E-02<br>1.98E-03 ± 3.73E-03	2.88E-03 ± 5.07E-03<br>1.44E-02 ± 6.11E-03	2.94E-03 ± 5.74E-03<br>1.43E-02 ± 3.86E-03	0.189
Methanol	-8.91E-03 ± 4.13E-03<br>-7.59E-03 ± 8.37E-03	1.39E-02 ± 4.44E-02<br>-1.69E-02 ± 4.89E-03	-1.24E-02 ± 5.40E-03<br>-2.02E-02 ± 9.38E-03	-7.57E-03 ± 4.57E-03<br>-1.35E-02 ± 5.25E-03	0.158
Taurine	1.39E-03 ± 2.12E-03<br>3.27E-03 ± 4.93E-03	8.97E-02 ± 1.62E-01<br>2.30E-03 ± 4.74E-03	-2.49E-03 ± 2.31E-03<br>1.03E-02 ± 5.66E-03	9.14E-04 ± 3.27E-03<br>6.37E-03 ± 6.14E-03	0.730
Glucose	4.27E-02 ± 9.10E-02<br>1.34E-02 ± 1.97E-01	4.64E-02 ± 2.44E-01<br>-9.40E-02 ± 8.51E-02	-2.06E-04 ± 1.25E-01<br>-1.86E-01 ± 2.33E-01	8.81E-02 ± 1.38E-01<br>-8.75E-02 ± 1.09E-01	0.190
Carnitine	5.18E-03 ± 1.34E-03<br>-1.96E-03 ± 1.05E-03	7.97E-04 ± 3.94E-03<br>5.94E-04 ± 1.54E-03	-2.39E-03 ± 1.53E-03<br>-8.95E-04 ± 3.24E-03	1.14E-03 ± 2.02E-03<br>-1.53E-03 ± 1.39E-03	0.115
Choline	1.05E-03 ± 8.28E-04<br>5.93E-04 ± 1.03E-03	-2.94E-04 ± 1.31E-03<br>4.61E-04 ± 6.95E-04	-7.19E-04 ± 3.82E-04<br>-8.82E-04 ± 1.23E-03	5.67E-04 ± 6.28E-04<br>3.88E-04 ± 9.32E-04	0.926
Dimethyl-sulfone	2.47E-04 ± 2.54E-04<br>-6.04E-04 ± 3.50E-04	3.03E-04 ± 6.81E-04<br>-4.09E-04 ± 3.45E-04	4.03E-04 ± 5.13E-04<br>-3.28E-04 ± 3.99E-04	5.30E-04 ± 4.51E-04<br>-2.93E-04 ± 3.50E-04	**0.008**
Malonate	-1.96E-03 ± 1.20E-03<br>7.84E-04 ± 1.41E-03	2.16E-04 ± 3.17E-03<br>9.46E-04 ± 1.37E-03	-1.91E-03 ± 7.88E-04<br>2.20E-03 ± 3.30E-03	-2.28E-03 ± 1.17E-03<br>-5.98E-04 ± 1.03E-03	**0.026**
Ornithine	5.74E-04 ± 1.13E-03<br>1.64E-03 ± 1.13E-03	-2.26E-04 ± 2.19E-03<br>1.94E-03 ± 1.19E-03	-2.65E-03 ± 1.85E-03<br>2.18E-03 ± 2.38E-03	-8.80E-05 ± 1.16E-03<br>8.89E-04 ± 1.66E-03	**0.031**
Creatine	-8.69E-04 ± 1.14E-03<br>7.29E-05 ± 1.15E-03	-1.30E-04 ± 1.56E-03<br>-3.08E-05 ± 1.06E-03	-2.00E-03 ± 1.05E-03<br>-2.78E-04 ± 1.63E-03	-6.32E-04 ± 9.50E-04<br>-7.47E-04 ± 9.13E-04	0.414
Lysine	4.16E-03 ± 2.80E-03<br>-8.43E-03 ± 5.57E-03	6.66E-03 ± 1.19E-02<br>-6.46E-03 ± 7.08E-03	-6.27E-03 ± 4.12E-03<br>-1.15E-02 ± 1.29E-02	-1.64E-03 ± 3.63E-03<br>-1.73E-03 ± 6.34E-03	0.060
Asparagine	3.80E-04 ± 1.95E-03<br>2.38E-03 ± 6.78E-04	-1.06E-03 ± 3.02E-03<br>1.48E-03 ± 8.40E-04	1.35E-03 ± 2.83E-03<br>1.21E-03 ± 1.52E-03	-1.14E-03 ± 1.65E-03<br>2.44E-03 ± 1.64E-03	0.076
Dimethylglycine	1.49E-04 ± 1.66E-04<br>-3.33E-05 ± 6.12E-05	1.66E-04 ± 3.14E-04<br>1.53E-04 ± 9.99E-05	3.01E-04 ± 1.14E-04<br>1.02E-05 ± 1.08E-04	4.27E-05 ± 1.40E-04<br>-1.73E-05 ± 8.41E-05	0.202
Trimethylamine	4.10E-05 ± 3.23E-05<br>-1.42E-05 ± 6.55E-05	-6.35E-05 ± 1.36E-04<br>-1.28E-05 ± 6.24E-05	-2.47E-05 ± 7.00E-05<br>9.27E-05 ± 9.41E-05	-2.42E-05 ± 5.29E-05<br>8.56E-05 ± 5.05E-05	0.255
Aspartate	1.15E-03 ± 6.40E-04<br>6.76E-04 ± 5.80E-04	1.55E-04 ± 7.14E-04<br>5.86E-04 ± 6.65E-04	1.05E-03 ± 9.75E-04<br>6.04E-04 ± 8.57E-04	1.02E-03 ± 5.48E-04<br>3.82E-04 ± 1.40E-03	0.630
Sarcosine	1.51E-03 ± 1.21E-03<br>3.66E-05 ± 1.80E-04	1.02E-03 ± 1.19E-03<br>1.70E-04 ± 1.56E-04	1.84E-04 ± 2.12E-04<br>-2.41E-04 ± 1.89E-04	1.96E-04 ± 1.83E-04<br>1.85E-05 ± 1.92E-04	0.240
Dimethylamine	4.95E-05 ± 6.10E-05<br>-3.73E-05 ± 5.52E-05	-9.34E-06 ± 2.47E-04<br>-9.29E-05 ± 3.35E-05	-7.39E-05 ± 7.10E-05<br>-1.77E-05 ± 9.88E-05	-3.69E-05 ± 9.47E-05<br>-7.30E-05 ± 5.13E-05	0.460
Methionine	1.28E-03 ± 1.02E-03<br>1.60E-04 ± 8.42E-04	-3.05E-03 ± 6.08E-03<br>4.46E-04 ± 7.36E-04	-4.15E-03 ± 4.21E-03<br>7.36E-04 ± 1.16E-03	-2.95E-03 ± 2.65E-03<br>-6.15E-04 ± 5.03E-04	0.391
Citrate	8.17E-04 ± 1.98E-03<br>-1.94E-03 ± 2.21E-03	-5.69E-01 ± 9.87E-01<br>-1.64E-03 ± 1.51E-03	-7.62E-01 ± 6.59E-01<br>-1.01E-03 ± 1.92E-03	-5.29E-01 ± 4.91E-01<br>-1.41E-03 ± 8.61E-04	0.083
Glutamine	1.53E-03 ± 7.13E-03<br>-2.41E-03 ± 8.56E-03	2.23E-04 ± 1.69E-02<br>-5.55E-03 ± 7.41E-03	1.11E-02 ± 1.01E-02<br>-5.32E-03 ± 1.24E-02	2.18E-03 ± 9.44E-03<br>-3.50E-03 ± 7.32E-03	0.379
Succinate	4.44E-04 ± 2.87E-04<br>-1.19E-04 ± 2.23E-04	-4.05E-04 ± 4.69E-04<br>-3.27E-04 ± 2.16E-04	-4.01E-04 ± 3.30E-04<br>-1.03E-04 ± 4.61E-04	-1.73E-04 ± 2.24E-04<br>-6.08E-05 ± 2.32E-04	0.806
Acetone	-4.47E-03 ± 2.91E-03<br>-1.35E-03 ± 3.12E-03	-1.21E-02 ± 2.61E-02<br>-1.90E-03 ± 2.82E-03	-2.20E-02 ± 1.66E-02<br>-3.29E-03 ± 5.00E-03	-1.73E-02 ± 1.23E-02<br>-2.77E-03 ± 3.84E-03	0.196
Glutamate	-9.38E-03 ± 1.34E-02<br>4.68E-02 ± 2.27E-02	4.31E-02 ± 6.73E-02<br>-2.55E-02 ± 1.33E-02	-3.65E-03 ± 2.07E-02<br>2.66E-02 ± 4.27E-02	1.74E-02 ± 1.78E-02<br>9.00E-03 ± 2.33E-02	0.990
Proline	-9.95E-03 ± 4.85E-03<br>6.37E-02 ± 7.98E-02	-1.29E-02 ± 1.06E-02<br>2.47E-04 ± 6.92E-03	1.24E-03 ± 1.12E-02<br>4.67E-03 ± 1.19E-02	-8.53E-03 ± 4.64E-03<br>6.21E-02 ± 7.02E-02	0.075
Arginine	5.09E-03 ± 3.20E-03<br>2.51E-03 ± 3.17E-03	-1.89E-03 ± 8.18E-03<br>7.15E-03 ± 4.26E-03	4.06E-03 ± 3.84E-03<br>4.80E-03 ± 4.06E-03	3.25E-03 ± 3.43E-03<br>3.50E-03 ± 3.82E-03	0.375

Acetate	3.54E-03 ± 1.87E-03<br>7.25E-03 ± 6.75E-03	1.22E-02 ± 2.55E-02<br>1.15E-03 ± 2.36E-03	-1.60E-03 ± 9.53E-04<br>5.38E-03 ± 7.69E-03	1.24E-03 ± 2.73E-03<br>-3.05E-04 ± 1.82E-03	0.504
Alanine	1.39E-04 ± 7.88E-03<br>1.38E-05 ± 5.92E-03	-1.11E-02 ± 2.45E-02<br>-8.67E-03 ± 7.62E-03	-1.19E-03 ± 1.12E-02<br>5.24E-03 ± 1.30E-02	-1.14E-02 ± 7.35E-03<br>6.62E-03 ± 9.31E-03	0.360
2-Hydroxyisobutyrate	-1.25E-03 ± 8.24E-04<br>-7.30E-05 ± 4.63E-04	4.75E-03 ± 1.04E-02<br>-5.04E-06 ± 3.35E-04	-1.36E-03 ± 1.35E-03<br>2.63E-04 ± 1.07E-03	-6.26E-04 ± 9.05E-04<br>-2.79E-04 ± 4.51E-04	0.581
3-Hydroxybutyrate	-3.12E-03 ± 3.20E-03<br>-1.47E-03 ± 3.77E-03	-5.33E-03 ± 1.49E-02<br>1.16E-03 ± 2.22E-03	5.72E-04 ± 5.52E-03<br>-4.89E-04 ± 1.73E-03	6.67E-04 ± 3.96E-03<br>9.85E-04 ± 2.44E-03	0.866
Ethanol	-8.15E-03 ± 3.15E-03<br>1.57E-01 ± 1.77E-01	7.17E-01 ± 1.25E+00<br>-7.88E-04 ± 5.79E-03	3.27E-03 ± 9.76E-03<br>1.86E-02 ± 3.20E-02	-2.84E-03 ± 4.44E-03<br>-3.35E-04 ± 7.88E-03	0.574
2.3-Butanediol	-9.42E-03 ± 9.19E-03<br>2.75E-03 ± 1.47E-03	3.10E-03 ± 2.93E-02<br>3.19E-03 ± 1.95E-03	-1.24E-02 ± 1.24E-02<br>6.17E-03 ± 3.68E-03	-8.89E-03 ± 9.50E-03<br>1.39E-03 ± 1.49E-03	0.137
Methylsuccinate	3.77E-04 ± 2.31E-04<br>4.87E-04 ± 4.79E-04	1.84E-03 ± 2.77E-03<br>2.10E-04 ± 1.94E-04	1.60E-04 ± 1.92E-04<br>-8.74E-05 ± 1.71E-04	2.70E-04 ± 1.91E-04<br>1.56E-05 ± 1.48E-04	0.365
3-Hydroxyisobutyrate	-1.28E-04 ± 3.25E-04<br>2.90E-04 ± 5.51E-04	9.12E-03 ± 1.30E-02<br>-2.54E-04 ± 3.14E-04	-3.67E-04 ± 4.65E-04<br>-7.93E-05 ± 6.54E-04	-7.35E-05 ± 4.15E-04<br>-8.17E-05 ± 4.16E-04	0.707
Isoleucine	-4.50E-04 ± 8.58E-04<br>2.64E-03 ± 1.01E-03	5.68E-03 ± 1.23E-02<br>4.03E-03 ± 7.90E-04	-2.57E-04 ± 1.98E-03<br>1.42E-03 ± 1.24E-03	-8.90E-04 ± 8.62E-04<br>3.49E-03 ± 1.69E-03	**0.012**
Valine	-5.17E-03 ± 2.27E-03<br>2.55E-03 ± 2.48E-03	-6.75E-03 ± 5.31E-03<br>3.07E-03 ± 2.03E-03	-1.58E-03 ± 1.68E-03<br>1.44E-03 ± 2.37E-03	-3.65E-03 ± 1.77E-03<br>-7.63E-04 ± 2.40E-03	**<0.001**
2-Aminobutyrate	-9.31E-04 ± 5.34E-04<br>-2.61E-04 ± 5.38E-04	3.35E-03 ± 8.24E-03<br>4.07E-05 ± 3.19E-04	-5.77E-04 ± 8.61E-04<br>-5.84E-04 ± 6.08E-04	-2.82E-04 ± 4.41E-04<br>-4.41E-04 ± 4.71E-04	0.699
Leucine	1.41E-03 ± 2.27E-03<br>2.72E-03 ± 1.37E-03	-2.03E-03 ± 4.71E-03<br>3.88E-03 ± 1.88E-03	-4.92E-04 ± 1.91E-03<br>7.10E-04 ± 1.96E-03	1.43E-04 ± 1.53E-03<br>1.62E-03 ± 1.94E-03	0.065
